# Acute Eosinophilic Pneumonia: A Rare Complication of Daptomycin Therapy

**DOI:** 10.7759/cureus.6803

**Published:** 2020-01-28

**Authors:** Michael H Storandt, Abhishek Matta

**Affiliations:** 1 Internal Medicine, University of North Dakota School of Medicine, Grand Forks, USA; 2 Internal Medicine, University of North Dakota School of Medicine, Fargo, USA

**Keywords:** daptomycin, eosinophilic pneumonia

## Abstract

Daptomycin is a cyclic lipopeptide antibiotic with great efficacy targeting gram-positive cocci, including methicillin-resistant Staphylococcus aureus and vancomycin-resistant enterococcus. Acute eosinophilic pneumonia is a rare complication of daptomycin therapy, with a poorly understood etiology thought to involve the accumulation of the drug in pulmonary surfactant inducing inflammation. We present a 56-year-old male with a history of spinal epidural abscess being treated with intravenous daptomycin, who presented to the emergency department with fever, developed worsening shortness of breath, and was subsequently discovered to have eosinophilia of pulmonary secretions via bronchoalveolar lavage. Daptomycin was discontinued, and he underwent treatment with corticosteroids, resulting in full resolution of symptoms. Diagnosis of acute eosinophilic pneumonia requires a high degree of clinical suspicion. Identification may be further complicated by the fact that symptoms can present anywhere from days to weeks after starting the therapy. This complication is very responsive to treatment with corticosteroids and cessation of daptomycin, but recognition is essential. With an increasing use of daptomycin subsequent to continued emergence of antibiotic resistance, it is essential that physicians are aware of this rare complication of daptomycin therapy.

## Introduction

Daptomycin is a cyclic lipopeptide antibiotic, first approved by the Food and Drug Administration (FDA) in 2003, with great efficacy targeting gram-positive cocci, including methicillin-resistant *Staphylococcus aureus* and vancomycin-resistant enterococcus [1]. Indications for use include complicated skin and skin structure infections, *Staphylococcus aureus* bacteremia, and right-sided infective endocarditis, in addition to multiple off-label uses [2]. It is commonly used as an alternative to vancomycin for outpatient intravenous antibiotic therapy due to its convenient once-daily dosing [2]. Serious complications of daptomycin therapy include myopathy/rhabdomyolysis, anaphylaxis, and acute eosinophilic pneumonia (AEP) [3]. 

AEP is a rare complication of daptomycin use, first reported by Hayes et al. in 2007, who described that a 60-year-old male developed eosinophilic pneumonia concurrent to daptomycin therapy for aortic valve endocarditis that subsequently improved after cessation of the therapy and intravenous methylprednisolone was initiated [4]. Kim et al. identified 7 definite, 13 probable, and 38 possible cases between 2004 and 2010 via review of the FDA Adverse Event Reporting System [5]. Its etiology is poorly understood but is thought to involve the accumulation of the drug within pulmonary surfactant, inducing an inflammatory response [6]. Multiple diagnostic criteria have been established, including one by the FDA and another by Solomon and Schwarz [7-8]. Treatment involves cessation of the drug, and in a majority of cases, intravenous steroids [6,9]. We present a case of this rare complication of daptomycin therapy.

## Case presentation

A 56-year-old male with a twenty pack-year smoking history quitting 15 years prior to presentation, with a history of spinal epidural abscess being treated with daptomycin (6 mg/kg/intravenous dose every 24 hours), presented to the emergency department with a fever of 104° F. The patient had a history of recurrent epidural abscesses, requiring multiple surgical washouts, following past lumbar diskectomy. Previous cultures were positive for two strains of *Staphylococcus epidermidis*, and the patient was started on a 6-week course of IV daptomycin approximately 2 weeks prior to the current presentation.

**Figure 1:**
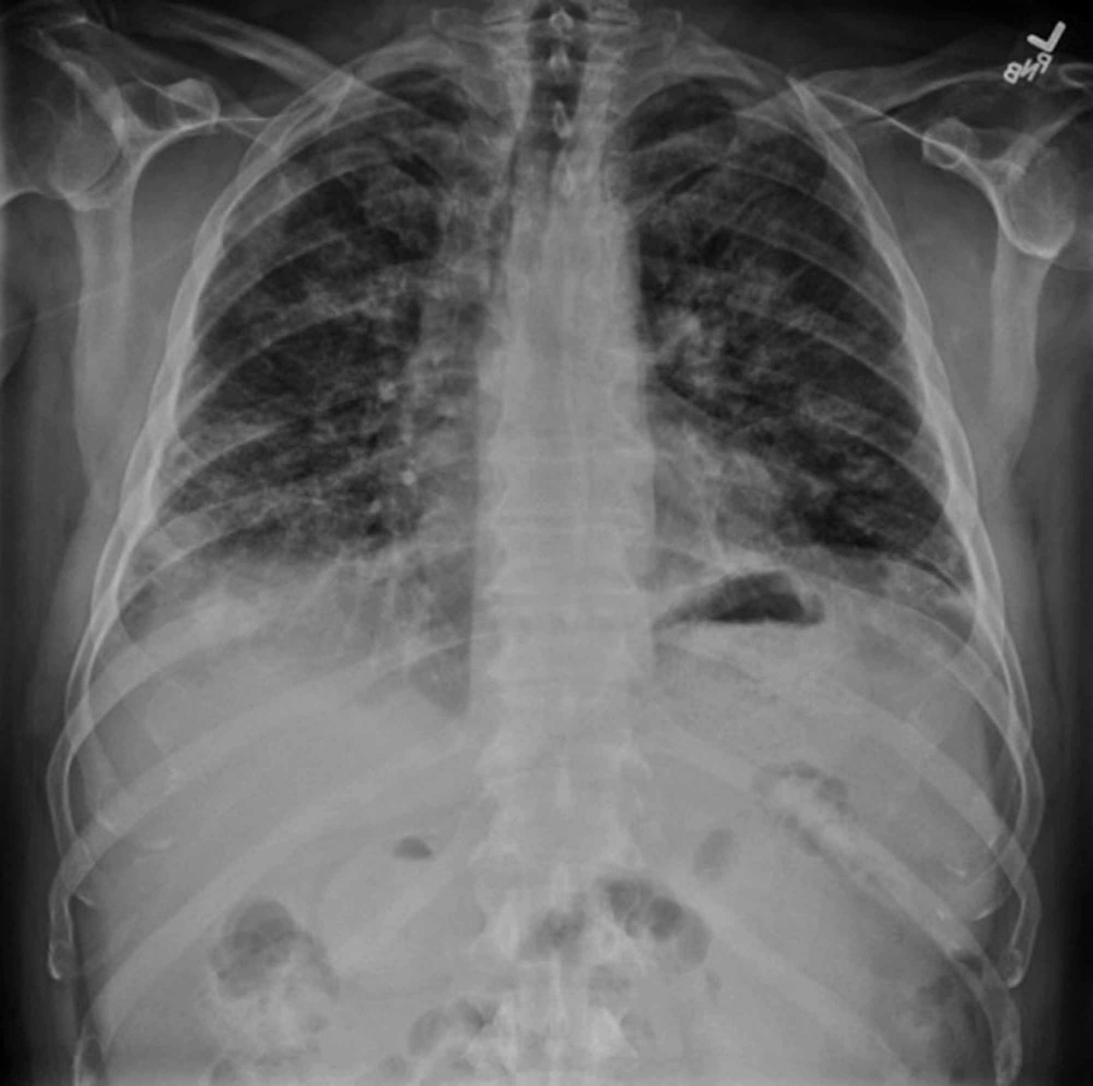
Chest X-ray demonstrating bilateral patchy interstitial infiltrates

The patient was admitted and started on empiric vancomycin and cefepime as fever was thought to be due to continued infection at the surgical site. Initial MRI re-demonstrated a ventral epidural fluid collection that was decreased in size from his previous MRI. Due to continued fever on hospital day 2, a chest X-ray (CXR) was ordered, which demonstrated patchy interstitial pneumonic infiltrates bilaterally (Figure 1), although the patient did not report pulmonary symptoms at this point in time, and the patient was started on empiric treatment with azithromycin. Subsequent consult via pulmonology noted that the patient’s CXR, in conjunction with eosinophilia noted on CBC, could indicate eosinophilic pneumonia, likely secondary to recent daptomycin use. Bronchoscopy with bronchoalveolar lavage was performed on hospital day 6. Fluid analysis revealed 46% eosinophils, 33% neutrophils, 11% lymphocytes, 2% basophils, and 9% macrophages/monocytes. Work-up of BAL for infection included *Mycobacterium tuberculosis *detection via PCR, *Nocardia* culture, *Aspergillus* PCR, and Beta-D-glucan assay, all of which were negative. Based on his clinical presentation, in addition to eosinophilia of pulmonary secretions, the patient was started on 40 mg intravenous methylprednisolone twice daily for the remaining four days of hospitalization for treatment of AEP. 

The patient was discharged on hospital day 9 with intravenous vancomycin, oral levofloxacin, an approximately 7-week oral steroid taper, and oral sulfamethoxazole-trimethoprim for pneumocystis pneumonia prophylaxis while taking the steroids. CXR 7 weeks after discharge demonstrated significant resolution of the interstitial lung opacities. At present, the patient has no residual pulmonary symptoms.

## Discussion

Acute eosinophilic pneumonia is a pulmonary condition characterized by eosinophilia of pulmonary secretions, which may be idiopathic or induced by various exposures, including drugs, infections, and smoking [10]. In the case of daptomycin-induced AEP, it is thought that the accumulation of the drug within a pulmonary surfactant plays a key role in the induction of an inflammatory reaction [6]. In reported cases, dosing is significantly varied, suggesting that the development of AEP may occur at any dose, however, among patients who develop AEP, those receiving higher doses had a shorter time to develop [6,9]. Additionally, elevated serum concentration due to chronic kidney disease may play a role in some cases, although a correlation between serum levels of daptomycin, and accumulation within pulmonary surfactant has not been definitively established [6]. In the present case, the patient had a normal renal function, with a creatinine of 0.96 mg/dL and blood urea nitrogen of 14 mg/dL. A serum concentration of daptomycin was not obtained. It is thought that as the drug accumulates in surfactant, it is phagocytosed by macrophages and presented via major histocompatibility complex (MHC) class 2, activating Th2 T-cells, which subsequently release IL-5 leading to eosinophil activation and chemotaxis [11]. This generates an inflammatory response causing damage to the lung parenchyma, however, the exact mechanism by which this occurs is not well understood [10].

Various diagnostic criteria exist for the diagnosis of daptomycin-induced AEP (Table 1). The FDA requires exposure to daptomycin, fever, dyspnea with increased oxygen requirement or requiring mechanical ventilation, new infiltrates on CXR or CT scan, bronchoalveolar lavage with >25% eosinophils, and clinical improvement with daptomycin withdrawal for definitive diagnosis [7]. Solomon and Schwarz have additionally proposed diagnostic criteria, but require reintroduction of daptomycin to demonstrate recurrence of eosinophilic pneumonia upon re-challenge, but this is not advised due to risk to the patient [8]. In addition to the diagnostic criteria, many patients present with peripheral eosinophilia, although this is only present in approximately 77% of patients, and therefore, is not required for diagnosis [9]. AEP may be difficult to recognize due to its delayed presentation. One systematic review found the mean duration of daptomycin therapy prior to the development of symptoms to be 2.8 ± 1.6 weeks, with one study reporting presentation of symptoms as late as 6 weeks into therapy [9,12]. In the presented case, the patient became symptomatic after 2 weeks of daptomycin therapy and met all diagnostic criteria as established by the FDA.

**Table 1:** Diagnostic criteria for daptomycin-induced eosinophilic pneumonia

	Food and Drug Administration [7]	Soloman and Schwarz [8]
Criteria	Concurrent exposure to daptomycin	Presence of eosinophilic pneumonia by diagnostic criteria (eosinophilic excess on lung biopsy or via bronchoalveolar lavage in setting of parenchymal infiltrates)
	Fever	Exposure to likely drug or toxin within recent time period
	Dyspnea with increased oxygen requirement or requiring mechanical ventilation	No other identifiable causes (parasites, fungal infection)
	New infiltrates on chest X-ray or CT scan	Clinical improvement upon cessation of suspected offending agent
	Bronchoalveolar lavage with >25% eosinophils	Recurrence of eosinophilic pneumonia upon re-challenge
	Clinical improvement with daptomycin withdrawal	

There is no established treatment regimen for AEP, but a majority of patients undergo treatment with corticosteroids [6,9]. However, there are reported cases of resolution by simply discontinuing daptomycin [13-15]. Prognosis of daptomycin-induced AEP is promising, with multiple literature reviews demonstrating the resolution of symptoms in all patients [6,9].

## Conclusions

Daptomycin is a relatively new antibiotic with increasing use, in part due to convenience for outpatient intravenous antibiotic therapy compared to vancomycin. Acute eosinophilic pneumonia is an uncommon complication of daptomycin therapy but is easily resolved with the detection and cessation of therapy. With the increased frequency of use, it is essential that physicians are aware of this rare complication of daptomycin therapy. In the above case, we present a patient who developed eosinophilic pneumonia following 2 weeks of daptomycin therapy for the treatment of recurrent spinal epidural abscesses, who subsequently improved following the cessation of therapy with the initiation of intravenous steroids and has no residual pulmonary symptoms at present.
